# Prevalence of undiagnosed diabetes and pre-diabetes among hypertensive patients attending Kiambu district Hospital, Kenya: a cross-sectional study

**DOI:** 10.11604/pamj.2015.22.286.7395

**Published:** 2015-11-24

**Authors:** Nkatha Meme, Samuel Amwayi, Ziporrah Nganga, Esther Buregyeya

**Affiliations:** 1 Field Epidemiology Training Program, Ministry of Health, Kenya; 2 Jomo Kenyatta University of Science and Technology, Kenya; 3 Makerere University, College of Health Sciences School of Public Health, Uganda

**Keywords:** Diabetes, pre-diabetes, glycated haemoglobin, hypertensive patients

## Abstract

**Introduction:**

Hypertension (HTN) and diabetes mellitus (DM) are two common non-communicable diseases (NCDs) that are closely linked: one cannot be properly managed without attention to the other. The aim of this study was to determine the prevalence of undiagnosed diabetic and pre-diabetic states that is abnormal glucose regulation (AGR) and factors associated with it among hypertensive patients in Kiambu Hospital, Kenya.

**Methods:**

We conducted a cross-sectional study from February 2014 to April 2014. Hypertensive patients aged ≥18 attending the out-patient medical clinic were included in the study. Pregnant and known diabetic patients were excluded. Data was collected on socio-demographics, behavior, and anthropometrics. Diabetes status was based on a Glycated Haemoglobin (HbA1C) classification of ≥6.5% for diabetes, 6.0-6.4% for pre-diabetes and ≤6.0% for normal. AGR was the dependable variable and included two diabetic categories; diabetes and pre-diabetes.

**Results:**

We enrolled 334 patients into the study: the mean age was 59 years (Standard deviation= 14.3). Of these patients 254 (76%) were women. Thirty two percent (107/334; 32%) were found to have AGR, with 14% (46) having un-diagnosed DM and 18%(61) with pre-diabetes. Factors associated with AGR were age ≥45 (OR = 3.23; 95% CI 1.37 ≥ 7.62), basal metabolic index (BMI) ≥ 25 Kg/m^2^ (OR= 3.13; 95% CI 1.53 - 6.41), low formal education (primary/none)(OR= 2; 95%CI 1.08 - 3.56) and family history of DM (OR = 2.19; 95%CI 1.16 - 4.15).

**Conclusion:**

There was a high prevalence of undiagnosed AGR among hypertensive patients. This highlights the need to regularly screen for AGR among hypertensive patients as recommended by WHO.

## Introduction

In 2012 non-communicable diseases (NCDs) were responsible for 68% of deaths globally and almost three quarters of these deaths (28 million), occurred in low- and middle-income countries [1]. It is well known that hypertension (HTN) and diabetes mellitus (DM) are two common NCDs that are closely linked: one cannot be properly managed without attention to the other [2–4]. The presence of hypertension substantially increases the risk of morbidity from several diseases, particularly cardiovascular diseases andDM. Therefore targeted screening for identification of risk factors, early detection and timely treatment can significantly reduce morbidity and mortality related to these NCDs [3]. The World Health Organization (WHO) recommends that patients presenting with HTN should have a cardiovascular risk assessment, including blood glucose testing [1, 4].

Majority of diabetic patients go through a pre-diabetes phase for several years [5–8], during which there is an opportunity to identify them and initiate timely prevention. Pre-diabetes is an intermittent stage of overt diabetes where the blood glucose level is higher than the normal value but not high enough to meet the criteria for the diagnosis of diabetes mellitus [7, 9]. It is characterized by a fasting plasma glucose (FPG) level of 6.1-6.9mmol/l and/or from an Oral Glucose Tolerance Test (OGTT) as a 2-hour post-load plasma glucose level of 7.8-11mmo/l (Impaired Glucose Tolerance (IGT) [10] or glycated haemoglobin A1C (HbA1C) level of 5.7-6.4% [11] or 6.1 - 6.4% [12–14]. Pre-diabetes increases the risk of diabetes mellitus in hypertensive patients and both undiagnosed pre-diabetes and diabetes are associated with diabetic complications [15, 16].

Abnormal glucose regulation (AGR) is a term used to definethe two glycometabolic states, that is diabetes mellitus and pre-diabetes [7]. In most studies AGR assessment is based on random blood sugar, FPG, OGTT. However, studies have shown that use of HbA1c is a convenient alternative test [17–19] as it is highly standardized, exhibits low intra-individual variation, can be obtained at any time, requires no patient preparation, and samples are relatively stable at room temperature after collection [20, 21]. There is also a strong correlation between average plasma glucoseand HbA1C in predicting diabetes development in patients with cardiovascular disease such as hypertension, coronary artery disease and stroke [22–27].

According to WHO recommendations hypertensive patients are to undergo diabetes screening based on their risk profile [28]. The American Diabetes Association (ADA) recommends that adults at normal risk for diabetes undergo screening every 3 years and adults at high-risk based on a family history of the disease, hypertension, overweight or obesity, or other factors of diabetes undergo screening every 1 to 2 years [29]. However the optimal universally acceptable interval for diabetes screening of healthy adults or adults with hypertension or hyperlipidemia is not known according the U.S. Preventive Services Task Force (USPSTF) [30]. Clinical judgment and risk profile of the patientshould determine when to screen individual patients for diabetes [29, 30]. One-third of all people with diabetes may be undiagnosed and more than 60% of newly diagnosed diabetics are unaware of their condition until complications appear [3, 31–33]. According to data from the 2011 Minnesota behavioral risk factor surveillance system, 30% of hypertensive adults had not received a blood glucose test within the previous 3 years. Among them, 10.7% had pre-diabetes and 19.6% had undiagnosed diabetes [34]. A cross sectional study conducted among hypertensive patients at Mulago National Referral Hospital, Uganda in 2012 found AGR in 237 (74%) patients - 50% were pre-diabetic and 24% were undiagnosed diabetic [35]. Failure to screen for AGR among hypertensive patients and lack of awareness about the importance of screening by health providers may indicate missed opportunities for early detection, clinical management, and prevention of diabetes [34].

There is scarcity of documented data on prevalence of AGR among high risk groups, such as hypertensive patients in clinical settings in Kenya. Therefore, the aim of this study was to determine the prevalence of undiagnosed diabetic and pre-diabetic states and factors associated with AGR among hypertensive patients in Kiambu Hospital, Kenya.

## Methods

### Design and study setting

We conducted a cross sectional study from February 2014 to April 2014 among hypertensive patients attending the out-patient medical clinic in Kiambu District Hospital, in Kiambu County, Kenya. Kiambu Sub-county is a predominantly rural area but is experiencing a rise inthe urban population because it borders Nairobi, the capital city of Kenya to the north. Kiambu Hospital is a high volume district hospital that offers general in-patient and out-patient services with a catchment population of about 101,596 and bed capacity of 316 and 67 cots.The hospital holds an out-patient medical clinic once a week. Patients enrolled into this clinic comprise of patients with HTN and other medical conditions. Patients are seen on appointment scheduled on average every 2-3 months depending on their medical condition for routine checkup and drug refill. At every visit routine tests include blood pressure (BP) reading and basal metabolic rate (BMI) calculations. Diabetes testing is done at the initial visit. Any subsequent diabetes tests are requested based on the patients presenting symptoms at the time of the visit and discretion of the clinician.

### Study population and participant selection

Hypertensive patients aged 18 years and above who were not known diabetic patients attending an out-patient medical clinic were included in the study. Pregnant and known diabetic patients were excluded from the study. Eligible participants were asked to participate in the study as they visited the medical out-patient clinic.

### Sample size calculation and sampling

Using the Cochran formula [36] our calculated sample size was 334 hypertensive patients, assuming a 74% prevalence of undiagnosed diabetes among the hypertensive patients [35] with a 95% confidence intervaland 10% adjustment for non-response. Patientswereselected usingsystematic sampling, with every third hypertensive patient enrolled into the study.

### Data collection and study variables

We utilized a structured questionnaire adapted from the WHO STEPS instrument for collecting surveillance data for non-communicable diseases (NCD's) for this study [37]. The questionnaire was pre-tested and administered by a trained medical healthworker. Social demographics and behaviouralvariables such as age, sex, level of education, occupation, tobacco use, alcohol consumption, physical activity and diet were collected.Physical activity was assessed by asking participants if they undertook “vigorous-intensity activities” (e.g.lifting heavy loads, cutting firewood, digging, construction work, etc) and “moderate-intensity activities” (e.g. brisk walking, carrying light loads, milking cows, washing clothes, riding a bicycle, light recreational activities,etc) in a typicalweek. Pictorial showcards were used to describe these activities to participants. Time spent on these activities in a typical week was recorded. Participants were classified into those that met the WHO minimum recommendations for physical activity (at least 75 minutes of vigorous-intensity, or 150 minutes of moderate-intensityactivities per week) and those that did not [38]. In addition, history of anti-hypertensive medication, prior diabetes evaluation and family history of DM among the 1st degree relatives, [39] was collected. The outcome variable was AGR.

Physical assessments included blood pressure, height, weight, waist and hip circumference measurements and calculation of body mass index (BMI) and waist-hip ratio, as detailed in a study by Ayah et al [40]. Body mass index (BMI), calculated as weight (kg)/height (m^2^) was used as a measure of total body obesity: BMI < 18.5 was recorded as underweight; 18.5-24.9 as normal; 25-29.9 as overweight; and >30 as obese. Waist-hip ratio (waist circumference/hip circumference) was used as a measure of central/abdominal obesity: >0.85 in women and >0.95 in men.The waist circumference was measured using a flexible tape-measure. Measurement was made in the mid-axillary line midway between the last rib and the superior iliac crest and the recording was at the point of normal expiration. The hip measurement was made using a flexible tape-measure placed horizontally at the point of maximum circumference over the buttocks. Measurement was made to the nearest 0.5 cm. Height was measured with the subject standing upright against a wall on which was affixed a height measuring device. Measurements were made with the subject barefoot, standing with the back against the wall and head in the Frankfort position with heels together. The subject was asked to stretch to the fullest and then exhale. When appropriately positioned, the measurements were taken to the nearest 0.5 cm. Weight measurements were taken on a pre-calibrated weighing scale. Subject was weighed while dressed in light clothing and barefoot. Measurements were made to the nearest 0.5 kg [40].

Blood pressure (BP) was measured using a mercury sphygmomanometer blood pressure device (Reisterdiplomat-presameter^®^). The Seventh Report of the Joint National Committee guideline for hypertension measurement and management was used [41]. Three intermittent readings were taken with the BP machine cuff placed mid - arm and an average of the last two readings used for the study. Hypertension was defined as systolic BP ≥ 140 mmHg and/or diastolic BP ≥ 90 mm Hg or use of prescribed anti-hypertensive medication [2].

A blood sample was collected from each study participant by drawing 2ml by venipuncture into an Ethylenediaminetetraaceticacid (EDTA) bottle and the HbA1C measured using a National Glycoheamoglobin Standardization Program (NGSP) analyser (Roche Cobas^®^Integra HbA1c Analyser) in a NGSP accredited laboratory.Classification of abnormal glucose regulation (AGR) was based on the revised WHO criteria and studies suggesting HbA1c cut-off points with high specificity and sensitivity for pre-diabetes screening [12, 13, 32]. HbA1C was grouped into diabetic HbA1c ≥6.5%, pre-diabetic HbA1c of 6.1% - 6.4% and non-diabetic HbA1c <6.1%. AGR included two categories 1) those with diabetes (participants with HbA1c ≥6.5%) and 2) those with pre-diabetes (participants with a HbA1c of 6.1% - 6.4%).

### Data management and analysis

Data was entered into version 3.5.4 of Epi-Info (CDC, Atlanta, USA) and analysed. Specific descriptive variables were analysedusing frequencies, proportions and means to describe the social-behavioural and clinical characteristics. Bivariate analysis using Chi-square testwere performed. Bivariate and multivariable analysis using logistic regressionwas used to explore the factors associated with AGR. An association was considered significant at P < 0.05and 95% confidence intervals (CI). Variables that had a P value ≤0.2 at bivariate level were included in the multivaria blelogistic analysis model with backward elimination to find independent factors that were associated with AGR.

### Ethical considerations

The study protocol was reviewed and approved by the Kenyatta National Hospital (KNH) Ethical Review Committee and administrative clearance sought from Kiambu District Hospital administration. We obtained written informed consent from each study participant after giving a detailed explanation of the purpose, risks and benefits of the study. Participants were contacted and given their results. Those with an AGR category of newly diagnosed diabetes were referred to the diabetic clinic for further management and follow-up while those with pre-diabetes were put on lifestyle interventions to reduce the risk of progression to diabetes.

## Results

### Socio-behavioralcharacteristics of participants

We enrolled 334 hypertensive patients into the study: the mean age was 58.6 years (Standard deviation = 14.3). Seventy six percent (254/334) were women, 38.9% (133/334) had no formal education, Table 1. Almost all the participants (97%; 323/334) reported not smoking tobacco at the time of the interview and 70% (232/334) had never consumed alcohol in their life time. Over half (56.3%; 188/334) of the respondents had truncalobesity and 40% (134/334) were found obese according to their BMI, Table 2. Almost a quarter of the respondents (21.3%-71/334) reported a history of familial diabetes.

**Table 1:** Socio-demographic characteristics of hypertensive adults at Kiambu Hospital, Kenya by glucose regulation status (N = 334)

	Diabetic n (%)	Pre-Diabetic n (%)	Normal n (%)	All Participants N (%)
**Sex**				
Male	7 (15.2)	13 (21.3)	60 (26.4)	80 (24)
Female	39 (84.8)	48 (78.7)	167 (73.6)	254 (76)
**Age group in years**				
25 - 44	3 (6.5)	4 (6.6)	55 (24.2)	62 (18.6)
45- 64	16 (34.8)	25 (41)	99 (43.6)	140 (41.9)
65 - 84	23 (50)	32 (50.8)	66 (29.1)	120 (35.9)
85 and above	4 (8.7)	1 (1.6)	7 (3.1)	12 (3.6)
**Level of education completed**				
None	27 (58.7)	31 (50.8)	75 (33.1)	133 (39.8)
Primary	9 (19.6)	19 (31.7)	72 (31.7)	100 (29.9)
Secondary (4 years)	9 (19.6)	7 (11.5)	67 (29.5)	83 (24.9)
University/college(2-4 years)	1 (2.2)	4 (6.6)	13 (5.7)	18 (5.4)
**Ethnicity**				
Kikuyu	43 (93.5)	59 (96.7)	208 (91.6)	310 (92.8)
Others	3 (6.5)	2 (3.3)	19 (8.4)	24 (4.2)
**Marital status**				
Currently married	23 (50)	36 (59)	145 (63.9)	204 (61.1)
Divorced	2 (4.3)	3 (4.9)	8 (3.5)	13 (3.9)
Never married	3 (6.5)	4 (6.6)	34 (15)	41 (12.3)
Widowed	18 (39.1)	18 (29.5)	40 (17.6)	76 (22.8)
**Occupation**				
Small scale farmer	23 (50)	31 (51)	75 (33)	129 (39)
Unemployed	10 (22)	11 (18)	48 (21)	69 (21)
Skilled formal	3 (7)	3 (5)	21 (9)	27 (8)
Informal	10 (22)	8 (13)	61 (27)	109 (33)

**Table 2:** Behavioral and anthropometric characteristics of hypertensive patients at Kiambu Hospital, Kenya by glucose regulation status (N = 334)

	Diabetic n (%)	Pre-Diabetic n (%)	Normal n (%)	All Participants N (%)
**Currently smoke tobacco (N = 334)**				
Yes	2 (4.3)	0 (0)	9 (4)	11 (3.3)
No	44 (95.7)	61 (100)	218 (96)	323 (96.7)
**Smoked tobacco in the past (N = 323)**				
Yes	2 (4)	4 (6.6)	22 (10)	28 (8.4)
No	42 (96)	57 (93.4)	197 (90)	296 (88.9)
**Ever consumed Alcohol (N = 334)**				
Yes	13 (28.3)	14 (23)	75 (33)	102 (30.5)
No	33 (71.7)	47 (77)	152 (67)	232 (69.5)
**Adequate Physical Activity**				
Yes	25 (54)	39 (64)	148 (65)	212 (64)
No	21 (46)	22 (36)	79 (35)	122 (36)
**Truncal Obesity**				
Yes	30 (65.2)	32 (52.5)	126 (55.5)	188 (56.3)
No	16 (34.8)	29 (47.5)	101 (44.5)	146 (43.7)
**BMI kg/m2**				
Underweight (<18.5)	0 (0.0)	1 (1.6)	1 (0.4)	2 (0.6)
Normal (18.5-24.9)	5 (10.9)	5 (8.2)	62 (27.3)	72 (21.6)
Overweight (25-29.9)	18 (39.1)	21 (34.4)	87 (38.3)	126 (37.7)
Obese (≥ 30)	23 (50.0)	34 (55.7)	77 (33.9)	134 (40.1)
**On prescribed HTN medication (N = 297)**				
Yes	38 (88.4)	54 (96.4)	178 (89.9)	270 (90.9)
No	5 (11.6)	2 (3.6)	20 (10.1)	27 (9.1)
**Blood sugar measured in the past 12 months**				
Yes	21 (70.0)	29 (69)	87 (66.9)	137 (67.8)
No	9 (30.0)	13 (31)	43 (33.1)	65 (32.2)
**Family Member with diabetes**				
Yes	13 (28.3)	17 (27.9)	41 (18.1)	71 (21.3)
No	33 (71.7)	44 (72.1)	181 (79.7)	258 (77.2)
Don't Know	0 (0.0)	0 (0)	5 (2.2)	5 (1.5)

### Prevalence of AGR and determinants

More than a third (32%; 107/334) of the participants had AGR (diabetes and pre-diabetes). Fourteen percent (46/334) of the participants were newly diagnosed as diabetic (HbA1C of ≥ 6.5%); while 18% (61/334) were pre-diabetic (HbA1C of 6.1% - 6.4%). Sixty eight percent (137/334) of the participants reported ever been screened for DM in the hypertension clinic. On bivariate analysis AGR was significantly associated withage ≥ 45 years (Odds Ratio (OR) = 4.57; (95%CI 2.00 - 10.42), BMI ≥ 25 Kg/m^2^ (OR= 3.35; CI 1.68 - 6.67), lowformal education (primary/none) (OR 2.23; 95%CI 1.27 - 3.86) and history of a first degree relative with DM (OR = 2; 95%CI 1.11- 3.16). Having a controlled blood pressure below 140/90 mmHg was protective AGR (OR = 0.59; 95%CI 0.38-0.95), Table 3. On multivariate analysis, age ≥ 45 (OR = 3.23; 95% CI 1.37 - 7.62), BMI ≥ 25 Kg/m^2^ (OR= 3.13; 95% CI 1.53 - 6.41), low formal education (primary/none) (OR= 2; 95%CI 1.08 - 3.56) and history of a first degree relative with DM (OR = 2.19; 95%CI 1.16 - 4.15) were significantly associated with AGR, Table 3. Controlled blood pressure was significantly associated with normal blood sugar (OR = 0.59; 95% CI 0.36 - 0.97).

**Table 3:** Factors associated with Abnormal Glucose Regulation (AGR) among participants, Kiambu Hospital, Kenya

Variable	AGR N (Col%)	Normal N (Col%)	Unadjusted OR (95%CI)	Adjusted OR (95%CI)	P Value
**Age** ≥ **45 years**					
Yes	100 (94)	172 (75)	4.57 (2.00 - 10.42)	3.23 (1.37 - 7.62)	**0.0074**
No	7	55			
**Level of Education**					
None/primary	86 (80)	147 (65)	2.23 (1.27 - 3.86)	2.0 (1.08 - 3.56)	**0.027**
Secondary/Tertiary	21	80			
**BP(SBP or DBP)controlled**					
Yes	51 (48)	137 (60)	0.59 (0.38 - 0.95)	0.59 (0.36 - 0.97)	**0.037**
No	56	90			
**BMI**					
≥ 25kg/m2	96 (90)	164 (72)	3.35 (1.68 - 6.67)	3.13 (1.53 - 6.4)	**0.0017**
< 25kg/m2	11	63			
**First degree relative with DM**					
Yes	25 (23)	30 (13)	2 (1.11 - 3.16)	2.19 (1.16 - 4.15)	**0.011**
No	82	197			
**Truncal Obesity**					
Yes	62 (57.9)	126 (55.5)	1.1 (0.69 - 1.76)	-	-
No	45	101			
**Gender**					
Female	37 (81)	167 (74)	1.56 (0.89 - 2.76)	-	-
Male	20	60			
**Employment**					
Formal	6 (7)	21 (12)	0.56 (0.22 - 1.45)	-	-
Informal	80	158			
**Marital status**					
Married	59 (55)	145 (64)	0.7 (0.44 - 2.76)	-	-
Not Married	48	82			
**Tobacco Use**					
Yes	8 (8)	30 (13)	0.53 (0.23 - 1.20)	-	-
No	99	197			
**Harmful alcohol consumption**					
Yes	80 (75)	152 (67	1.46 (0.87 - 2.45)	-	-
No	27	75			
**Adequate physical activity**					
Yes	64 (60)	148 (65)	0.79 (0.49 - 1.27)	-	-
No	43	79			
**Glucose level ever measured**					
Yes	72 (67)	130 (57)	1.53 (0.94 - 2.49)	-	-
No	35	97			
**Glucose level measured in past 12mths**					
Yes	50 (69)	87 (67)	1.12 (0.06 - 2.08)	-	-
No	22	43			

## Discussion

This study aimed to determine the prevalence of undiagnosed diabetic and pre-diabetic states and factors associated with AGR among out-patient hypertensive patients in a clinical setting in Kenya. More than a third (32%) of the study participants had AGR i.e. diabetes and pre-diabetes. Fourteen percent were newly diagnosed with DM, while 18% were pre-diabetic. Sixty eight percent of the participants reported ever been screened for DM in the hypertension clinic. Risk factors for AGR were being 45 years and above, BMI > 25 kg/m^2^, low formal education and history of familial diabetes. Controlled blood pressure reduced the risk of developing AGR.

Our study found a high prevalence of AGR (diabetes and pre-diabetes) among hypertensive patients in the clinical setting. This is higher than population based studies previously done in Kenya which have a prevalence rangeof 3.3%to 6% [40, 42]. Accumulating evidence reveals that AGR is common among patients with cardiovascular diseases in hospital settings. In Uganda, Mutebi et al screened 320 hypertensive patients and found 50% were pre-diabetic and 24% were undiagnosed diabetic [35]. Kidney et al demonstrated out of 3847 hypertensive patients in Minnesota 10.7% had pre-diabetes and 19.6% had undiagnosed diabetes [34]. The Euro Heart Survey on diabetes and the heart demonstrated that AGR is more common than normal glucose metabolism in patients with coronary artery diseases and hypertension as 36% had pre-diabetes and 22% had newly detected diabetes [43]. In Germany, Luders et al found out of 260 hypertensive patients 39% had impaired glucose tolerance and 12% had diabetes mellitus [44].

Our findings show that AGR is significantlyassociated with age over 45 years and BMI above 25. Similar findings of high BMI and older age were demonstrated in parts of the world such as Nigeria [5], Uganda [6, 35] and in Germany [44]. Having a low level of education was associated with AGRin our study. In European countries with lower levels of education have been used as predictors of DM as this is comparable and similar to social economic status [45–47]. Our study showed that a family history of diabetes was also a risk factor for glucose intolerance among hypertensives and similar findings were demonstrated by Mutebi et al [35] and Hilding et al [39]. Our study found patients with controlled blood pressure had reduced risk of developing AGR. A study done by the U.S. Preventive Services Task Force (USPSTF)evidenced that lowering blood pressure below conventional target blood pressure values reduces the incidence of clinically detected diabetes [30].

In contrast tobacco use, alcohol use and inadequate physical activity were not associated with AGR in our study and could be due to the small sample size of the study. The study found that tobacco use and harmful alcohol use was not common practice among the study participants as 97% did not smoke tobacco, 89% had never smoked tobacco in the past and 70% had never consumed alcohol. This finding in variation may have been due to the sample size and the fact that our study subjects were skewed towards a predominantly female population.

There is evidence that the prevalence of AGR is high among hypertensive patients with specific risk factors, therefore a strong justification for use of targeted diabetic screening as it offers the patients and healthcare providers an opportunity to modify long-term risk before serious complications occur [6, 8, 48, 49]. Patients with newly diagnosed DM will benefit from proper glycemic control and reduction of complications and those with pre-diabetes will benefit from strategies tailored to prevent or retard onset of diabetes This also reduces the health costs associated with management of their hypertensive condition and possible concurrent type 2 diabetes mellitus [5, 6]. Though WHO recommends that patients with HTN be screened for diabetes, there are no guidelines on how this screening should be carried out. Specifically, how often they should be screened and criteria for risk groups to be targeted. Therefore, there is need for the Ministry of Health to develop guidelines to guide frontline health workers on how to implement screening for DM and among patients with HTN in order to achieve early diagnosis and timely treatment/interventions to be able to reduce morbidity and mortality.

Limitations are that the study findings can't be generalized to the population as this was a hospital based cross-sectional study. The study didn't collect information on the duration of hypertension diagnosis and the specific antihypertensive medication used by participants. It is known that various antihypertensive drugs have different effects on glucose metabolism such as thiazide diuretics and B blockers [50, 51]. However a study in Nigeria showed that anti-hypertensive drugs that affected glucose metabolism didn't significantly affect the prevalence of pre-diabetic states in hypertensive patients [5, 52]. The study findings would have been strengthened by availability of dietary and socio-economic data. Our physical activity data may suffer from the limitation of recall bias and being self- reported, even though the STEPS questionnaire has been validated in different populations. The study was not able to determine if patients had heamoglobinopathies or were taking medication that would lead to an underestimation or overestimation of the HbA1c results, however HbA1C provides flexibility in DM testing as it can be performed in a non-fasting stateat any time of the day, and therefore maybe more convenient for both healthcare professionals and patients.

## Conclusion

There was a high prevalence of undiagnosed AGR among hypertensive patients on follow-up. This highlights the need to regularly screen for AGR among hypertensive patients as recommended by WHO [28]. Using targeted screening based on risk profile to determine diabetic status offers patients and healthcare providers an opportunity to modify long-term risk before serious complications occur.There is need to have clear guidelines to health workers on how screening for AGR can be implemented.

## References

[1] WHO (2014). WHO_Global Status Report on Non-Communicable Diseases 2014.

[2] WHO (2007). WHO-Prevention of cardiovascular disease?: guidelines for assessment and management of total cardiovascular risk.

[3] WHO (2010). WHO Global status report on noncommunicable diseases.

[4] Whitworth JA (2003). 2003 World Health Organization (WHO)/International Society of Hypertension (ISH) statement on management of hypertension. J Hypertens..

[5] Iloh GUP (2013). Risk factors of pre-diabetes among adult nigerians with essential hypertension in a resource-constrained setting of a primary care clinic in eastern Nigeria. Am J Heal Res..

[6] Mayega RW, Guwatudde D, Makumbi F, Nakwagala FN, Peterson S, Tomson G (2013). Diabetes and pre-diabetes among persons aged 35 to 60 years in eastern Uganda: prevalence and associated factors. PLoS One..

[7] Pétur Pétursson (2012). Aspects of Abnormal Glucose Regulation in Various Manifestations of Coronary Artery Disease.

[8] Chatterjee R, Narayan KMV, Lipscomb J, Jackson SL, Long Q, Zhu M (2013). Screening for diabetes and prediabetes should be cost-saving in patients at high risk. Diabetes Care..

[9] Unwin N, Shaw J, Zimmet P, Alberti KGMM (2002). Impaired glucose tolerance and impaired fasting glycaemia: the current status on definition and intervention. Diabet Med..

[10] WHO/IDF W health organization / I consultation (2006). Definition and diagnosis of diabetes mellitus and intermediate hyperglycemia. Production.

[11] ADA ADA (2012). Standards of medical Care in Diabetes. Diabetes Care.

[12] Zemlin A, Matsha T, Hassan M, Erasmus R (2011). HbA1c of 6.5% to diagnose diabetes mellitus? does it work for us? The Bellville South Africa study. PLoS One..

[13] Kumar P, Bhansali A (2010). Utility of glycated hemoglobin in diagnosing type 2 diabetes mellitus: a community-based study. J J Clin Endocrinol Metab..

[14] Nathan D (2009). International Expert Committee report on the role of the A1C assay in the diagnosis of diabetes. Diabetes Care..

[15] Singleton JR, Smith AG, Russell JW, Feldman EL (2003). Microvascular Complications of Impaired Glucose Tolerance. Diabetes..

[16] Sowers JR, Epstein M, Frohlich ED (2001). Diabetes, hypertension, and cardiovascular disease: an update. Hypertension..

[17] Wang J-S, Lee I-T, Lee W-J, Lin S-Y, Fu C-P, Lee W-L (2015). Comparing HbA1c, fasting and 2-h plasma glucose for screening for abnormal glucose regulation in patients undergoing coronary angiography. Clin Chem Lab Med..

[18] Rohlfing C, Little R (2000). Use of GHb (HbA1c) in screening for undiagnosed diabetes in the US population. Diabetes..

[19] Edelman D, Olsen MK, Dudley TK, Harris AC, Oddone EZ (2004). Utility of hemoglobin A1c in predicting diabetes risk. J Gen Intern Med..

[20] Cowie CC, Rust KF, Byrd-Holt DD, Gregg EW, Ford ES, Geiss LS (2010). Prevalence of diabetes and high risk for diabetes using A1C criteria in the US population in 1988-2006. Diabetes Care..

[21] Jia Q, Zheng H, Zhao X, Wang C, Liu G, Wang Y (2012). Abnormal glucose regulation in patients with acute stroke across China: prevalence and baseline patient characteristics. Stroke..

[22] Yu Y, Ouyang X-J, Lou Q-L, Gu L-B, Mo Y-Z, Ko GT (2012). Validity of glycated hemoglobin in screening and diagnosing type 2 diabetes mellitus in Chinese subjects. Korean J Intern Med..

[23] Alqahtani N, Khan WAG, Alhumaidi MH, Ahmed YAAR (2013). Use of Glycated Hemoglobin in the Diagnosis of Diabetes Mellitus and Pre-diabetes and Role of Fasting Plasma Glucose, Oral Glucose Tolerance Test. Int J Prev Med..

[24] Exebio JC, Zarini GG, Vaccaro JA, Exebio C, Huffman FG (2012). Use of hemoglobin A1C to detect Haitian-Americans with undiagnosed Type 2 diabetes. Arq Bras Endocrinol Metabol..

[25] The Lewin group Studies on Hemoglobin A1c (HbA1c), Pre - Diabetes, and Diabetes.

[26] Selvin E, Steffes M, Zhu H (2010). Glycated hemoglobin, diabetes and cardiovascular risk in nondiabetic adults. N Engl J Med..

[27] Nathan D, Turgeon H, Regan S (2007). Relationship between glycated haemoglobin levels and mean glucose levels over time. Diabetologia..

[28] WHO/ISH (2003). 2003 World Health Organization (WHO)/International Society of Hypertension (ISH) statement on management of hypertension. Hypertens (Internet).

[29] American Diabetes Association ADA Diabetes Management Guidelines A1C Diagnosis ∣ NDEI (Internet).

[30] USPSTF the USPSTFA for HR and QRM (2008). Screening for Type 2 Diabetes Mellitus in Adults: US - Preventive Services Task Force Recommendation Statement. Ann Intern Med (American College of Physicians).

[31] IDF 2012 (2012). IDF Diabetes Atlas Update.

[32] WHO WHO 2011 (2011). Use of (HbA1c) in the Diagnosis of Diabetes Mellitus: Abbreviated Report of a WHO Consultation.

[33] Charfen MA, Ipp E, Kaji AH, Saleh T, Qazi MF, Lewis RJ (2009). Detection of undiagnosed diabetes and prediabetic states in high-risk emergency department patients. Acad Emerg Med..

[34] Kidney RSM, Peacock JM, Smith SA (2014). Blood glucose screening rates among Minnesota adults with hypertension, Behavioral Risk Factor Surveillance System, 2011. Prev Chronic Dis..

[35] Mutebi E, Nakwagala FN, Nambuya A, Otim M (2012). Original Article Undiagnosed diabetes mellitus and impaired glucose tolerance among hypertensive patients in Mulago Hospital, Kampala, Uganda.

[36] Cochran WG (1977). Sampling Techniques.

[37] WHO WHO (2008). WHO STEPS Instrument (Core and expanded).

[38] WHO WHO (2010) Global Recommendations on Physical activity for Health.

[39] Hilding A, Eriksson A-K, Agardh EE, Grill V, Ahlbom A, Efendic S (2006). The impact of family history of diabetes and lifestyle factors on abnormal glucose regulation in middle-aged Swedish men and women. Diabetologia..

[40] Ayah R, Joshi MD, Wanjiru R, Njau EK, Otieno CF, Njeru EK (2013). A population-based survey of prevalence of diabetes and correlates in an urban slum community in Nairobi, Kenya. BMC Public Health..

[41] Chobanian A, Bakris G, Black H (2003). Seventh report of the joint national committee on prevention, detection, evaluation, and treatment of high blood pressure. Hypertension..

[42] World Health Organization (2011). World Health Organization. Noncommunicable disease country profiles, Kenya.

[43] Bartnik M (2004). The prevalence of abnormal glucose regulation in patients with coronary artery disease across EuropeThe Euro Heart Survey on diabetes and the heart. Eur Heart J..

[44] Lüders S, Hammersen F, Kulschewski A, Venneklaas U, Züchner C, Gansz A (2005). Diagnosis of impaired glucose tolerance in hypertensive patients in daily clinical practice. Int J Clin Pract..

[45] Agardh EE, Sidorchuk A, Hallqvist J, Ljung R, Peterson S, Moradi T (2011). Burden of type 2 diabetes attributed to lower educational levels in Sweden. Popul Health Metr..

[46] Shang X, Li J, Tao Q, Li J, Li X, Zhang L (2013). educational level, obesity and incidence of diabetes among Chinese adult men and women aged 18-59 years old: an 11-year follow-up study. PLoS One..

[47] Sacerdote C, Ricceri F, Rolandsson O, Baldi I, Chirlaque M-D, Feskens E (2012). Lower educational level is a predictor of incident type 2 diabetes in European countries: the EPIC-InterAct study. Int J Epidemiol..

[48] Pastakia SD, Ali SM, Kamano JH, Akwanalo CO, Ndege SK, Buckwalter VL (2013). Screening for diabetes and hypertension in a rural low income setting in western Kenya utilizing home-based and community-based strategies. Globalization and Health..

[49] Van den Donk M, Sandbaek A, Borch-Johnsen K, Lauritzen T, Simmons RK, Wareham NJ (2011). Screening for type 2 diabetes: lessons from the addition-Europe study. Diabet Med..

[50] Rizos C, Elisaf MS (2014). Antihypertensive drugs and glucose metabolism. World J Cardiol..

[51] Eleftheriadou I, Tsioufis C, Tsiachris D, Tentolouris N, Stefanadis C (2011). Choice of antihypertensive treatment in subjects with pre-diabetes: is there a dream after the navigator. Curr Vasc Pharmacol..

[52] Essien OE, Peters EJ, Udoh AE, Ekott JU, Odigwe CO (2007). Prevalence and pattern of abnormal glucose tolerance in adult Nigerians with primary hypertension. Niger J Med..

